# The effect of combined loading on the behavior of micropiled rafts installed with inclined condition

**DOI:** 10.1007/s11356-022-21327-2

**Published:** 2022-06-22

**Authors:** Ahmed Elsawwaf, Ashraf Nazir, Waseim Azzam

**Affiliations:** Structural engineering department, Faculty of Engineering, Tanta University, Tanta, Egypt

**Keywords:** Micropiled raft, Finite element, Combined load, Lateral load capacity, Inclination angle, Load sharing ratio

## Abstract

One of the major disadvantages of micropiles is their low lateral stiffness and flexural rigidity due to the small diameter. This limitation can be handled in current practice, by installing the micropile with inclined condition or providing a steel casing. Additional steel casings will increase the lateral load capacity of micropiles but increase the project cost as well. Thus, inclination of micropile which is relatively simple and cheap is recommended. In this paper, a comprehensive numerical analysis is conducted on the behavior of micropiled rafts installed with inclined condition under combined vertical and lateral loading. A FEM calibrated against full-scale axial and lateral field tests is used to conduct the analysis. The soil profile is soft clay soil underlain by a layer of dense sand. The study investigates the impact of several parameters which are as follows: magnitude of vertical loading, reinforcement type, inclination angle of micropiles, and number of inclined micropiles. The study reveals that increasing vertical loads causes continuous decrease in the lateral load capacity of micropiled rafts. When all micropiles installed are inclined, the positively inclined micropiles carry 79–86% of the total lateral load carried by micropiles, whereas the negatively inclined ones carry 14–21%. Inclined micropiles offer greater lateral load sharing ratio (α_h_) than that of vertical ones, largest at *θ* = 45°. The effect of micropile reinforcement on improving the lateral performance is low compared to the effect of micropile inclination angle.

## Introduction

**Fig. 1:**
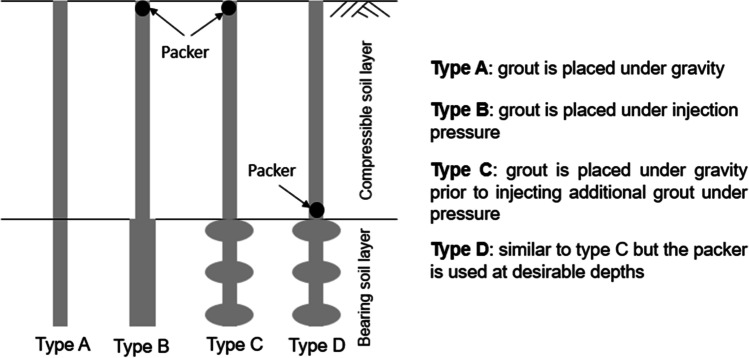
Micropile classification based on method of grouting

A micropile is essentially a small diameter cast in situ bored pile. Its diameter is typically in the range of 100–300 mm. In North America, micropiles were first used in 1973 through several underpinning applications in the New York and Boston areas and their use has been rapidly growing ever since. Based on their construction methods, micropiles are classified into four categories by FHWA (2005) as described in Fig. 1. Type A: the grout is concreted without any injection pressure. Type B: injection pressure is used in pouring the grout into the hole and typically ranges from 0.5 to 1 MPa. Type C: the grout is poured first under gravity head. Then, after the hardening of the poured grout, additional grout is poured using a sleeved pipe at a pressure of at least 1 MPa. Type D: similar to Type C but a packer can be used at specific depths inside the sleeved pipe (FHWA 2005; Kim et al. 2018). Using pressurized grout in the construction process of micropiles offers some advantages such as densifying the surrounding soil (especially for coarse-grained soil) and enhancing its shear strength. Since the grout is poured under pressure, it penetrates the soil offering an increased micropile section (Alnuaim et al. 2016). Practically, all structures are often subjected to lateral loads due to wind, wave loading, ship impact, etc. When considering onshore structures, lateral load can reach 15% of the applied vertical load, whereas it exceeds 30% of the applied vertical load in case of any marine structure (Rao et al. 1998; Subanantharaj Palammal and Senthilkumar 2018). Therefore, it is significant to assess the lateral behavior of micropiled rafts under combined loading. However, one of the major disadvantages of micropiles is their low lateral stiffness and flexural rigidity due to the small diameter. This limitation can be handled in current practice, by installing the micropile with inclined condition or providing a steel casing. Additional steel casings will increase the lateral load capacity of micropiles but increase the project cost as well. Thus, inclination of micropile which is relatively simple and cheap is recommended. To the knowledge of the authors, there are no guidelines on the lateral response of micropiled rafts installed with inclined condition under combined vertical and lateral loading. Even the studies related to their response under pure static lateral load are also very limited. Hence, in this paper, an attempt is taken to assess the performance of inclined micropiled rafts under combined loading through finite element modelling.

### Prime objectives of the study

The soil profile used in this study is soft clayey soil underlain by a layer of dense sand. An attempt has been taken to investigate the impacts of magnitude of vertical loading (F_v_), micropile reinforcement, inclination angle of micropiles (θ), and number of inclined micropiles, on micropiled raft performance under combined vertical and lateral loading. The performance is investigated in terms of the lateral response of the micropiled raft, vertical and lateral load sharing ratios between the micropiles and the raft, the lateral load carried by each individual micropile in the group, and the percentage of increase in the lateral load capacity.

## Background

The performance of the micropiled raft foundation is completely different from that of surface raft and similar to that of piled raft. Thus, the design concept of piled rafts under vertical loading can be adopted when analyzing micropiled rafts, where applied loads are shared by both the raft and micropiles. One of the well-known simplified methods that can be used in analysis is Poulos-Davis-Randolph (PDR) method which is based on the studies by Poulos and Davis (1980) and Randolph (1994). It can be used to assess the vertical load-carrying capacity of a piled raft considering the number of installed piles. Alnuaim et al. (2018) evaluated the availability of PDR method for micropiled rafts considering the relatively small diameter of micropiles compared to conventional piles. It was found that the PDR method can be adopted to analyze micropiled rafts with very stiff rafts. However, an adjustment factor was suggested in case of micropiled rafts with flexible rafts.

Regarding the vertical performance of micropiles and micropile groups, a number of studies have been performed through full-scale field tests. Jeon and Kulhawy (2001) conducted 21 full-scale field tests on pressure-grouted micropiles. The vertical load capacity of the micropile was significantly different from the drilled conventional pile due to the grouting pressure effect on the state of stresses in the surrounding soil. Han and Ye (2006) conducted load tests on a square raft (1.5 m × 1.5 m) supported by four micropiles. It was found that the load carried by the micropiles was about 70 to 86% of the additional vertical load applied to the micropiled raft. Wang et al. (2021) performed full-scale axial compression tests on Type A micropiles and waveform micropiles. Moreover, a micropiled raft consisting of 2 × 2 micropiles and a central waveform micropile was loaded vertically. The shear keys along the depth of waveform micropiles resulted in the enhancement of bearing capacity to be 1.5 times than that of Type A micropiles.

Experimental laboratory investigation was also conducted by many researchers on vertically loaded micropiled rafts and micropiles. For example, Tsukada et al. (2006) evaluated the mechanism which enhances the bearing capacity of a spread footing due to reinforcement with micropiles. Hwang et al. (2017) performed model tests and a numerical analysis to investigate the bearing capacity of a micropiled raft on medium dense sand and clayey silt which represent general shear failure and punching shear failure respectively. It was found that the effective installation of micropiles can enhance the bearing capacity by about 1.5–2.0 times than that of a surface raft. Borthakur and Dey (2018) investigated the vertical load carrying capacity of micropile group on highly plastic soft clay soil with cohesion in the range of 18–20 kPa. The load-carrying capacity increased with increasing the micropile spacing as well as their number.

FEA was also used by many researchers as an effective technique to study micropiles and micropile groups. Farouk (2009) found that the tension ultimate capacity of a single micropile tends to be lower than compressive ultimate capacity by about 7 to 12.5%. El Kamash and Han (2017) observed that vertical displacement of an existing surface raft underpinned by micropiles decreases with decreasing the initial pressure ratio and the increase of micropile length. Kim et al. (2018) conducted a series of numerical analyses to investigate the load carrying and load sharing behaviors of inclined micropiled rafts. Alnuaim et al. (2018) studied the vertical performance of micropiled rafts in soft clay. The tolerable bearing pressure of micropiled raft was 100% greater than that of isolated raft. El Kamash et al. (2022) conducted a numerical analysis to study the influence of consolidation on foundations underpinned by micropiles in soft clay. They stated that increasing time of consolidation causes higher settlement of the foundation and less skin friction along the depth of micropiles.

**Fig. 2:**
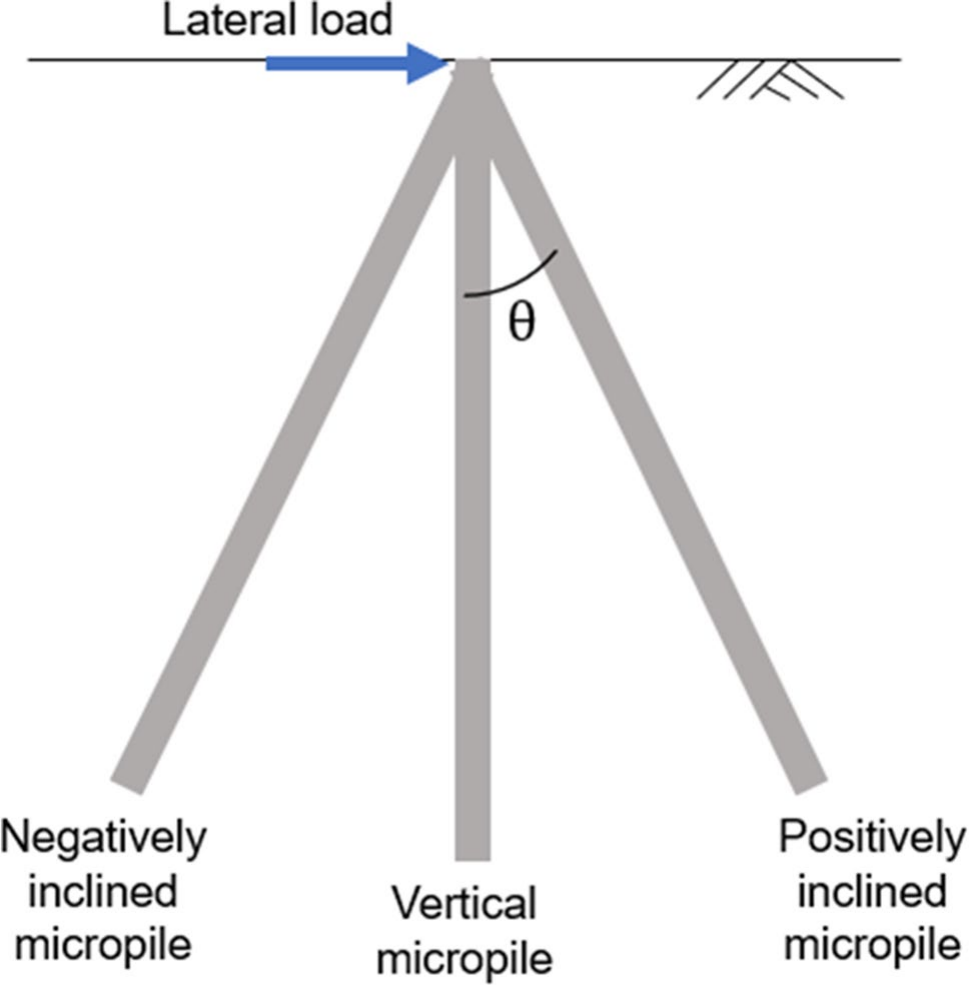
Negatively and positively inclined micropiles

Regarding the lateral performance of micropiles, numerous studies were conducted to assess the performance of vertical micropiles under pure lateral loading. For example, Teerawut (2002) performed field lateral load tests on vertical micropiles with different diameters installed in sand soil of different relative densities. The stiffness of the *p*–*y* curves increased as the pile diameter increased especially in dense sand. Abd Elaziz and El Naggar (2015) conducted two monotonic and six cyclic lateral loading tests on single micropiles in stiff to very stiff silty clay. A numerical analysis was also conducted. It was concluded that the micropile lateral capacity should be evaluated after careful consideration of the micropile connectivity into the pile cap. Kyung and Lee (2018) conducted a parametric study on the lateral load–carrying capacity of micropiles in order to assess the effect of micropile inclination and load direction. The study included model load tests, finite-element analyses, and full-scale field tests. The performance for both single and group micropiles was investigated. Moreover, the micropile mechanism of lateral load carrying was observed through the FEA. The study mainly depicted that lateral load capacity of inclined micropiles was found to increase with increasing the batter angle up to 30° for negatively inclined micropiles, whereas an opposite trend was observed for positively inclined micropiles (See Fig. 2). Sharma and Hussain (2019) performed a parametric study on the lateral behavior of inclined micropiles through model testing. The ultimate lateral capacity was found to be greater for the micropiles with a 15° and 30° negative inclination compared to the vertical and positively inclined piles. Malik et al. (2021) assessed the influence of installing confining micropiles around footings on the bearing capacity of sand. The study stated that installing inclined micropiles around footings could enhance their lateral load capacity. From all the above-mentioned studies, it is observed that only vertical loads or lateral loads were considered in the analysis of micropiles or micropile groups. The studies related to inclined micropiled rafts are limited as well. Thus, this paper aims to study the effect of combined vertical and lateral loading on inclined micropiled rafts. Moreover, a number of previous studies assessed the lateral response of vertical micropiles, but in a single soil layer. Hence, an attempt has been taken in the present study to consider the possible effect of soil stratum in the lateral response of inclined micropiled rafts.

## Finite element modelling

### Numerical model

**Fig. 3:**
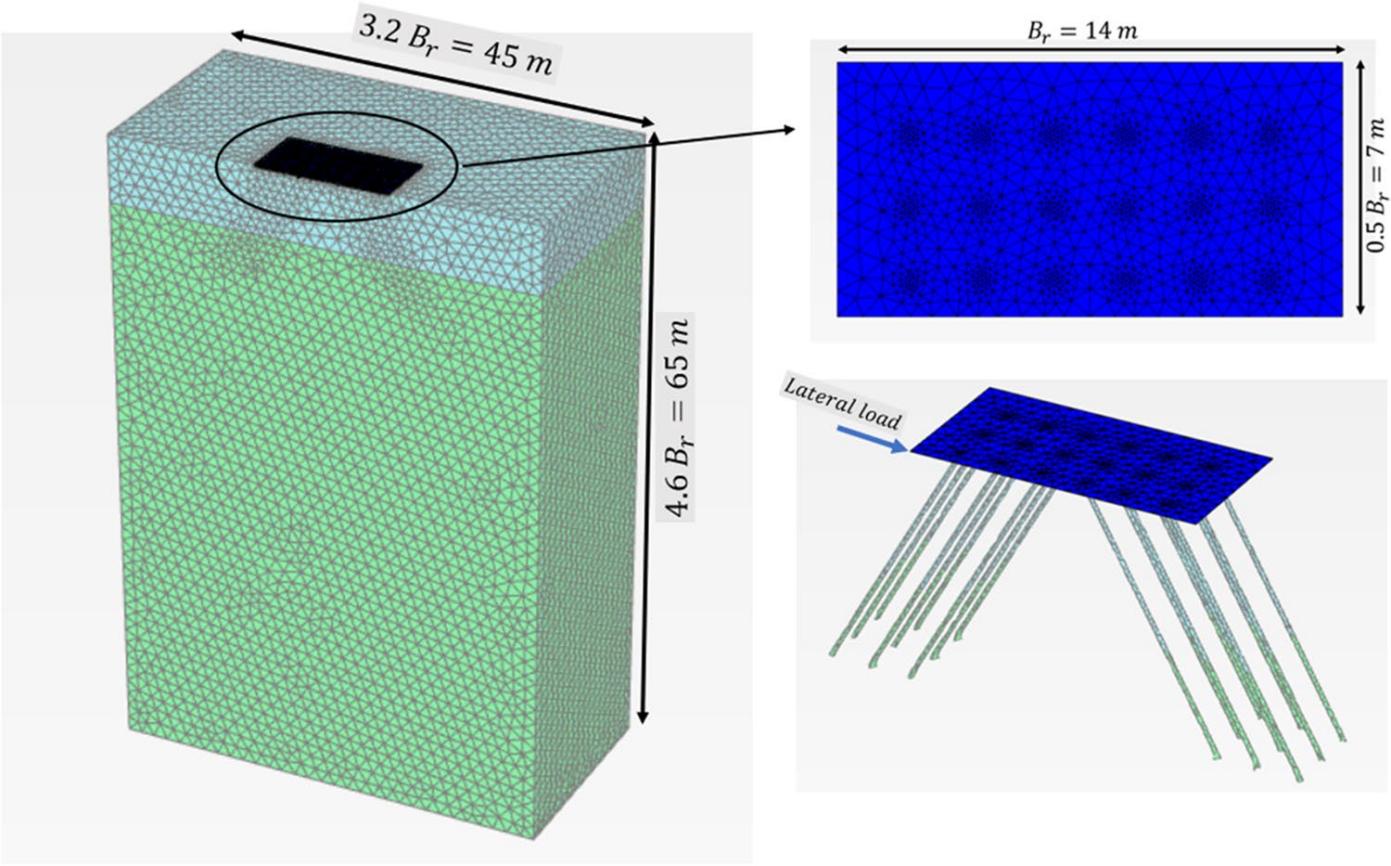
3D FEM used in the analysis and mesh pattern

The 3D model used to carry out the analysis was established using the computer program PLAXIS 3D. The validity of the FEM was checked by using the results of full-scale field loading tests and comparing them with those obtained from the FEA. The advantage of the symmetry across the *x*-axis was taken and a half of the micropiled raft foundation was modelled to decrease the computation time. Based on results of mesh sensitivity analysis, an appropriate size of the elements and location of mesh boundaries were adopted in order to minimize their effect on the calculated response. The horizontal side boundary was kept 3.2 *B*_*r*_ (where *B*_*r*_ is the raft width) and the vertical side boundary was kept 4.6 *B*_*r*_ as shown in Fig. 3. 3D 10-node tetrahedral elements were used to model the soil and micropiles while the raft foundation was modelled using 6-node triangular plate elements. In order to assure high accuracy of the results, denser mesh was used at locations where high stress concentration was expected (e.g., raft base, micropile base, and micropile side surface). The raft and micropiles were assumed to be linear elastic materials considering the mechanical properties (elastic modulus, and Poisson’s ratio). The Mohr-Coulomb model (elastic perfectly plastic behavior) was chosen for simulating the behavior of the soil. The Mohr-Coulomb model requires conventional soil parameters including unit weight, cohesion, friction angle, dilation angle, and Poisson’s ratio. In finite element analysis, interface elements are used to simulate the interaction between the micropile or the raft and the adjacent soil. These interface elements follow the Mohr-Coulomb failure criterion; as the shear stress reaches the maximum shear strength of the soil, slippage happens at the interface. In PLAXIS 3D, an interface reduction factor, *R*_*int*_, is used to model the interface element. *R*_*int*_ represents the strength of the interface element as a percentage of the shear strength of adjacent soil.

### Modelling of micropiles

**Fig. 4:**
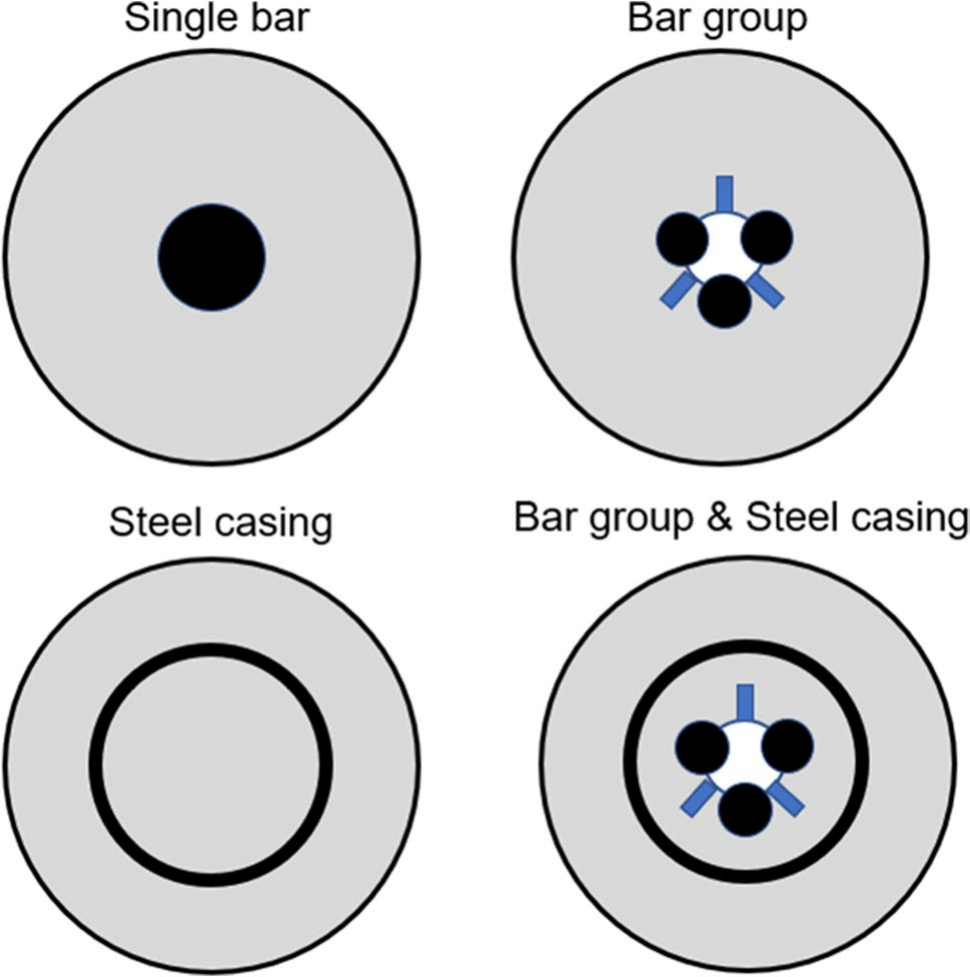
Different types of micropile reinforcement

In order to ensure that the micropile behavior is simulated in an accepted manner, its composition and construction method must be taken into consideration when modelling of micropiles. First, the large portion of steel area installed in micropiles induces high elastic modulus compared to conventional reinforced concrete cast in place piles. Figure 4 presents different cross sections of micropiles which are commonly used in practice. Reinforcement could be a single reinforcing bar, a group of reinforcing bars or a steel tube casing. In order to help improve the lateral performance of micropiles, an additional steel casing can be installed around the steel reinforcing bar(s). In the current study, steel bar group and steel bar group and casing are adopted. The true simulation of these two types of reinforcement requires proper estimation of the micropile young’s modulus based on the reinforcement percentage. Hence, it was estimated in the study as an average weighted modulus using the relationship proposed by FHWA (2005) as

1 $${\mathrm{E}}_{\mathrm{micropile}}=\left({\mathrm{A}}_{\mathrm{grout}}\times {\mathrm{E}}_{\mathrm{grout}}+{\mathrm{A}}_{\mathrm{steel}}\times {\mathrm{E}}_{\mathrm{steel}}\right)/{\mathrm{A}}_{\mathrm{micropile}}$$

where E_micropile_, elastic modulus of the micropile; A_grout_, section area of grout; E_grout_, elastic modulus of grout; A_steel_, section area of steel; E_steel_, elastic modulus of steel; and A_micropile_, section area of the micropile. According to the (FHWA 2005), the use of E_grout_, 31,000 MPa for confined grout, and E_grout_, 23,000 MPa for unconfined grout can provide reasonable results for micropiles.

Second, *R*_*int*_ value is expected to be high to simulate the rough surface condition for micropiles of Type B, Type C, and Type D, since these micropile construction techniques cause the soil surrounding the micropiles to densify (Alnuaim et al. 2016). Third, the pressurized grout for micropiles of Type B, Type C, and Type D causes a high confining pressure to the soil around the micropile, which would increase the lateral earth pressure coefficient (*K*_*s*_) (Farouk 2009; Alnuaim et al. 2018; Kim et al. 2018). Using the proper value of *K*_*s*_ is significant, since it greatly affects the vertical load capacity of micropile which is evaluated based on skin friction along the micropile depth according to the following relationship:

2 $${\mathrm{Q}}_{\mathrm{micropile}}={\upsigma}_{\mathrm{v}}^{\prime }.{\mathrm{K}}_{\mathrm{s}}.\tan \left({\varnothing}^{\prime}\right).\uppi .\mathrm{d}.{\mathrm{L}}_{\mathrm{b}}$$

where Q_micropile_, vertical load capacity of micropile; ${\upsigma}_{\mathrm{v}}^{\prime }$, effective vertical stress; K_s_, lateral earth pressure coefficient; ∅^′^, internal friction angle; d, micropile diameter; and L_b_, bond length.

### Comparison and validation of the model

The developed numerical model was validated in two stages. In the first stage, the results of full-scale field axial loading tests of a vertical micropile group Type C were compared with those obtained from the FEA. Then, in the second stage, the results of full-scale field lateral loading tests of an inclined group micropile Type C were compared with those obtained from the FEA. The details for both processes are presented in the following sub-sections.

### Case -1: field axial loading test of a vertical micropile group Type C

The validation process was conducted by comparing the estimated load-deformation behavior with the measured one by Kyung et al. (2017). The test site was located at Gochang city in Korea where layers of silty sands and clay were observed. Table 1 shows basic soil properties at the test site. A vertical group of 4 micropiles with spacing 1.26 m was installed. They were connected to a 2.52 m x 2.52 m square raft which was 1 m in thickness. The micropiles were 9.00 m in length and 0.165 m in diameter. Reinforcement used was a 65-mm-diameter steel rod (15.5% of area cross section) and the Type C grouting technique was used in the micropile installation, where gravity grouting was first poured, followed by pressurized grouting. Steel casing was placed within the upper 6 m.

**Table 1:** Soil properties in field test site at Gochang city

Parameter	Upper silty sand	Middle clay	Lower silty sand
Depth (m)	0–1	1–6	6–10
Unit weight (kN/m^3^), γ_t_	17.58	18.77	17.83
Angle of internal friction (^°^), ∅	28.64	0	33.52
Cohesion (kN/m^2^), S_u_	17.4	22.4	32.6

**Fig. 5:**
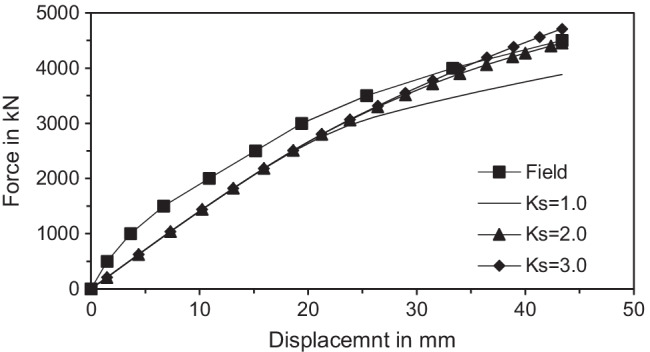
Comparison of numerical model results with full-scale field axial load tests of a vertical micropile group of Type C by Kyung et al. (2017)

The value of *E*_*mp*_ was 85 × 10^6^ kN/m^2^. The higher stiffness of micropiles was due to the large portion of steel area due to placing permanent upper steel casing. The average values of *E* of upper silty sand, middle clay, and lower silty sand were 5000 kN/m^2^, 14,000 kN/m^2^, and 14,000 kN/m^2^ respectively. Since the Type C grouting technique was used in the micropile construction, *R*_*int*_ tended to be high in the lower silty sand layer (uncased bond zone) and was assumed to be 0.95 (Alnuaim et al. 2016; Kyung and Lee 2018). Moreover, densification of the soil was expected which would in turn cause increase in the K_s_ value in the lower silty sand layer (uncased bond zone). Figure 5 shows the variation of the vertical load versus displacement behavior using different values of *K*_*s*_ for the lower silty sand layer. Since an increase in the *K*_*s*_ value was expected, the studied values began from a value of 1.00. Then, the *K*_*s*_ value was increased in successive runs till there was an acceptable matching between the estimated results and the field test results at a *K*_*s*_ value of (2.00 to 3.00). Similar approach was used by Farouk (2009) to evaluate the optimum value in *K*_*s*_.

### Case -2: field lateral loading test of an inclined micropile group Type C

The validation process was conducted by comparing the estimated load-deformation behavior with the measured one by Kyung and Lee (2018). The test site was located at Gochang city in Korea where layers of silty sands and clay were observed. Table 1 shows basic soil properties at the test site. An inclined group of 4 micropiles with spacing 1.26 m was installed. The inclination angle, *θ*, was 15°. The micropiles were connected to a 2.52 m x 2.52 m raft which was 1 m in thickness. They were 10.00 m in length and 0.165 m in diameter. Reinforcement used was a 65-mm-diameter steel rod (15.5% of area cross section) and the Type C grouting technique was used in the micropile installation, where gravity grouting was first poured, followed by pressurized grouting at an injection pressure of 1.3 MPa. Steel casing was placed within the upper 6 m.

**Fig. 6:**
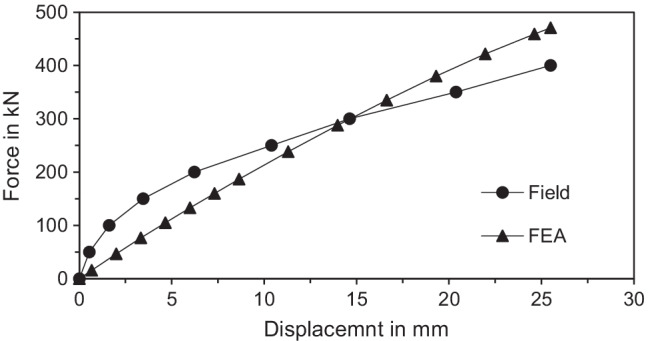
Comparison of numerical model results with full-scale field lateral load tests of an inclined micropile group of Type C by Kyung and Lee (2018)

The value of *E*_*mp*_ was 85 × 10^6^ kN/m^2^. The average values of *E* of upper silty sand, middle clay, and lower silty sand were 5000 kN/m^2^, 8000 kN/m^2^, and 8000 kN/m^2^ respectively. *R*_*int*_ tended to be high in the lower silty sand layer (uncased bond zone) and was assumed to be 0.95 (Alnuaim et al. 2016; Kyung and Lee 2018). Moreover, densifying of this soil layer was expected which would in turn cause increase in the *K*_*s*_ value which was taken 3.0. Figure 6 presents lateral load versus displacement behavior from numerical analysis compared to field test results obtained by Kyung and Lee (2018). With these values, a reasonable match with the full-scale field test results was achieved.

### Analysis outline

The numerical analysis was conducted through a number of stages. At the first stage, the initial stress field was calculated for the initial geometry configuration. At the second stage, the micropiled raft was installed. In the third stage, 16 vertical concentrated loads were applied at the top of the raft, and the magnitude of vertical loading was changed in order to assess its effect on the behavior of micropiled rafts. In the final step, a lateral displacement of 0.1 *d* (where *d* is the diameter of the micropile) was then applied at the micropiled raft to assess the combined loading effect (Deb and Pal 2019; Zormpa and Comodromos 2018).

### Model configuration and parametric study

**Fig. 7:**
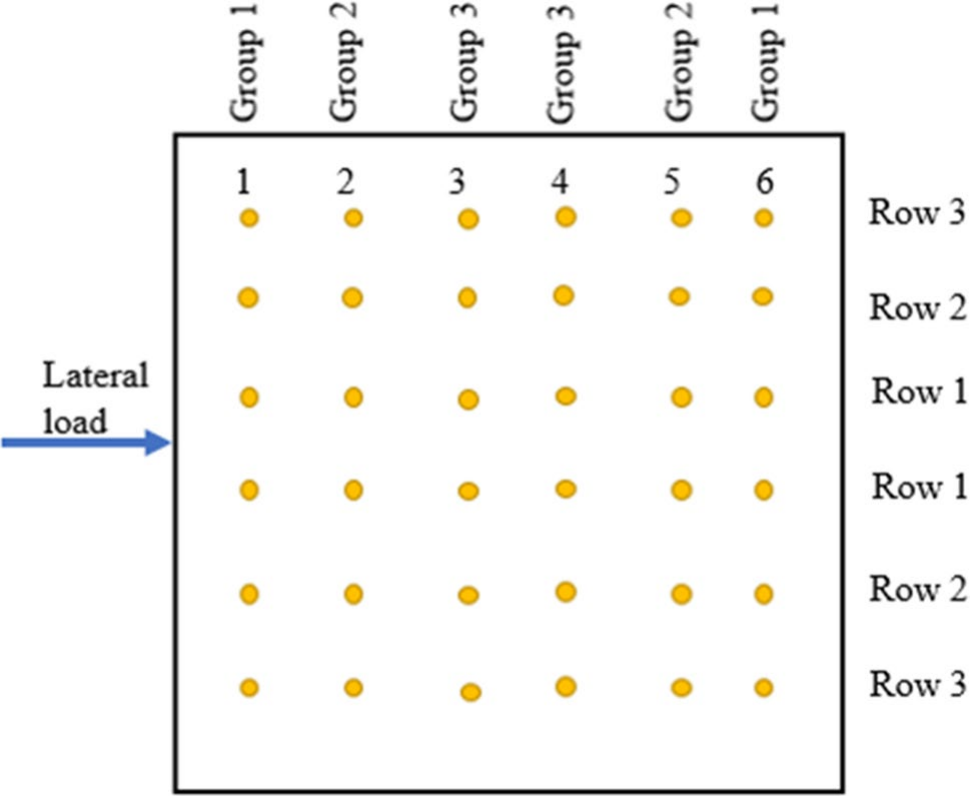
Layout of micropiles

In this study, a 14 m × 14 m × 0.8 m raft supported on 250 mm in diameter micropiles was used. A 6 × 6 micropile group was connected to the raft. The micropile length was 13 m and their spacing to diameter ratio was taken 8 as Juran et al. (2008) stated that the group effect gets significant when spacing to diameter ratio is smaller than 3–6. The soil profile was soft clay soil underlain by a layer of dense sand. Table 2 shows the input parameters of micropiles, and the raft used in the FEA. Vertically concentrated loads were adopted in the study. The studied parameters include magnitude of vertical loading (*F*_*v*_), micropile reinforcement type, inclination angle (*θ*), and number of inclined micropiles. In current practice, it is more common to install the micropiles vertically (not in an inclined condition), despite the ease of their installation at any angle. Installing several micropiles in an inclined condition may improve the lateral response and optimize the design of micropiled rafts. Therefore, an attempt has been taken to access the improvement of the lateral performance in case of installing combined inclined and vertical micropiles under the same raft. The micropiles in Group 1 only was first taken inclined (see Fig. 7). Then, the number of inclined micropiles was increased until all the 36 micropiles were inclined. Micropiles on the left-hand side were inclined in the negative direction, whereas the ones on the right-hand side were inclined in the positive direction compared to the lateral load direction (see Fig. 3). The detail program for the parametric study is presented in Table 3.

**Table 2:** Input parameters of micropiled raft used in FEM

Parameter	Raft	Micropile (bar group)	Micropile (bar group and casing)
Constitutive modelling	Linear elastic	Linear elastic	Linear elastic
Unit weight (kN/m^3^), γ_t_	24	24	24
Modulus of elasticity	22 × 10^6^ kPa	52 × 10^6^ kPa	85 × 10^6^ kPa
Steel reinforcement percentage	-	15.6%	29.0%
Poisson’s ratio, ν	0.15	0.15	0.15

**Table 3:** Parametric study

Vertical loading magnitude	Number of inclined micropiles	Inclination angle	Reinforcement
*F*_*v*_ = 0	Group 1 = 12 micropiles	0^°^, 15^°^, 30^°^, 45^°^	Bar group
*F*_*v*_ = 2298 kN	Group 1, 2 = 24 micropiles		Bar group with casing
*F*_*v*_ = 4595 kN	Group 1, 2, 3 = 36 micropiles		Bar group with casing
*F*_*v*_ = 6893 kN			

### Soil parameters

The Mohr-Coulomb model was chosen for simulating the behavior of the soil in the FE analyses. Table 4 summarizes input parameters used in the FEM for different soil layers. Bowles (1996) stated that the field values range of modulus of elasticity of soft clays and dense sands varies between 5000 and 25,000 kPa and between 50,000 and 81,000 kPa, respectively. Considering these field values ranges, the modulus of elasticity of the soil layers was selected. *R*_*int*_ was assumed 0.95 to simulate the rough surface condition for the micropiles of type c (Alnuaim et al. 2016; Kyung and Lee 2018). The most critical parameter that affects the shaft resistance of the micropiles is the lateral earth pressure coefficient *K*_*s*_. It depends on soil conditions, the pile geometry and material, and the pile construction method (Bowles 1996). Kim et al. (2018) stated that *K*_*s*_ could be in the range of 4–7 for sandy soils (the passive stress state). Olgun et al. (2019) stated that *K*_*s*_ could be between at rest earth pressure coefficient (*K*_*o*_) and passive earth pressure coefficient (*K*_*p*_ ). Therefore, choosing the proper *K*_*s*_ value for the soil layers in the current study is quite challenging due to the construction method of Type C micropiles. The validation process results of the FEM were utilized to choose the optimum value of *K*_*s*_ for sand and was taken 3.0 (see “Case -1: field axial loading test of a vertical micropile group Type C”). In case of the upper soft clay layer, *K*_*s*_ value was expected to be lower than that of sand. According to the (FHWA 2005), the shaft resistance of Type C micropiles in clay could reach 1.7 times the shaft resistance of Type A micropiles. This could indicate that *K*_*s*_ in case of Type C micropiles in clay could reach 1.7 K_o_. A value of 1.2 was adopted by Alnuaim et al. (2018) who studied micropiles of Type B in soft clay. Hence, a value of 1.4 is selected throughout this study which adopts micropiles of Type C.

**Table 4:** Input parameters of soil layers used in FEM

Parameter	Clay layer	Sand layer
Unit weight (kN/m^3^), γ_t_	17.5	20
Angle of internal friction (^°^), ∅	-	40
Dilation angle (^°^), ψ	-	10
Undrained cohesion (kPa), S_u_	25	-
Elastic modulus (kPa)	20,000	60,000
Poisson’s ratio, ν	0.4	0.3
Lateral earth pressure coefficient, *K*_*s*_	1.4	3.0
R_int_	0.95	0.95

### Applied vertical loads

**Fig. 8:**
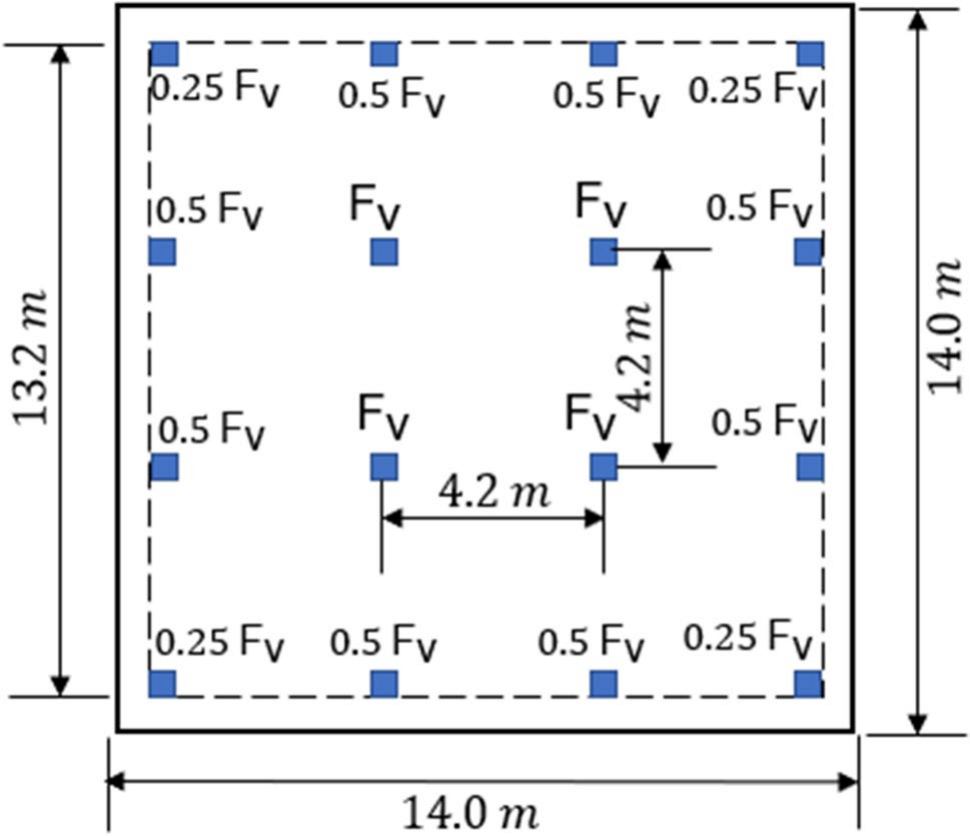
Layout of the columns on top of the raft

The loads of 16 columns of a multi-story building were considered in the study. The columns’ loads were evaluated according to the tributary area of each column. Thus, every internal column will transfer a vertical load of *F*_*v*_ to the raft, and every edge column will transfer 0.5 *F*_*v*_, whereas every corner column will transfer 0.25 *F*_*v*_. The vertical performance of micropiled rafts was analyzed after applying vertical loads and prior to applying the lateral load. In this stage, the performance-based design method was used. In this method, a tolerable settlement that will induce a functionality problem or maintenance issue for the building under working loads is specified. Then, the foundation system is designed such that the applied working loads cause settlement within the tolerable value (Roberts et al. 2011). In the current study, the maximum tolerable settlement was taken 7.5 cm, since the maximum overall settlement of piled rafts reported for several case histories was found to be between 60 and 100 mm (Alnuaim et al. 2016). Figure 8 shows the layout of the raft and columns.

## Analysis of results

### Pure vertical loads on micropiled raft foundation

**Fig. 9:**
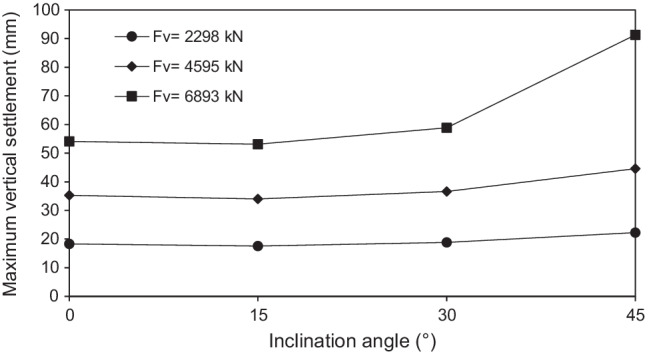
Maximum vertical settlement of micropiled rafts at various values of *F*_*v*_

All the studied cases were tested first under pure vertical loading to assess the vertical performance. Figure 9 shows the maximum actual vertical settlement of the raft compared to the assumed tolerable settlement of 7.5 cm, where all micropiles were installed inclined with steel casing around bar group. It can be seen that there is a slight decrease in the maximum settlement which does not exceed 4% when *θ* increases from 0 to 15° for all values of *F*_*v*_. However, the maximum settlement tended to increase when *θ* increases from 15 to 30°. This increase is 11% for *F*_*v*_ = 6893 kN, 7.5% for *F*_*v*_ = 4595 kN, and 7% for *F*_*v*_ = 2298 kN. When *θ* increases from 30° to 45°, the maximum settlement continuously increases and still, the rate of increase is the highest for *F*_*v*_ = 6893 kN compared to lower vertical loads. The maximum settlement of the raft at *θ* = 45° and *F*_*v*_ = 6893 kN is 9.13 cm which does not satisfy the assumed tolerable settlement (7.5 cm). These findings confirm that micropiled rafts offer the highest resistance to vertical loads at *θ* = 15° followed by a gradual decrease in the resistance with further increase of *θ*. The same trend was stated by Kyung et al. (2017).

### Evaluation of lateral response of micropiled raft

It is significant to specify a reasonable displacement level that corresponds to lateral load capacity due to the small diameter characteristics of micropiles. By considering a number of previous studies, it was found that Abd Elaziz and El Naggar (2015) selected displacement levels of 6.25, 12 mm, and 5% of micropile diameter to define the lateral load capacity of micropiles. Kyung and Lee (2018) specified the lateral load capacity of micropiles and micropiled rafts at a lateral displacement of 0.1 of the micropile diameter. Throughout the current study, the lateral load capacity is specified at 0.1 *d* lateral displacement which agrees with the trends of previous studies and is often used in practice.

### Effect of magnitude of vertical loading

**Fig. 10:**
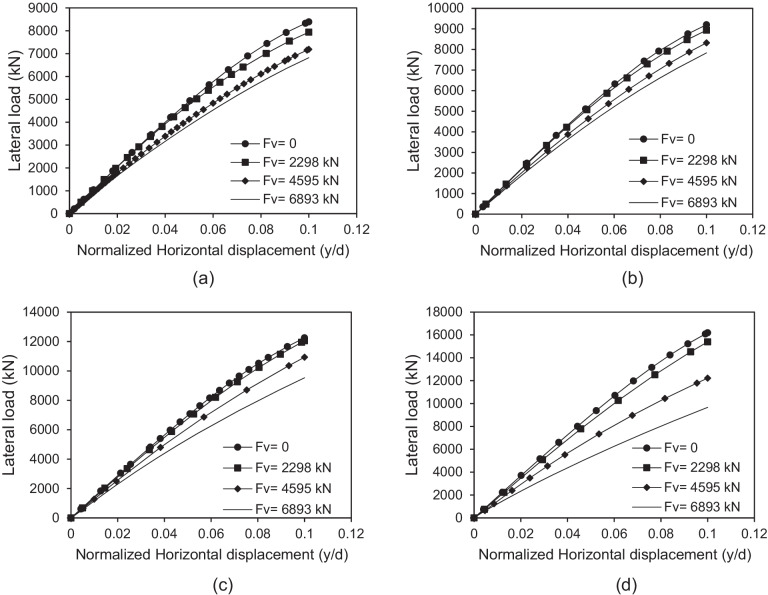
Lateral load displacement response of micropiled raft at various *F*_*v*_ values **a** 0° **b** 15° **c** 30° **d** 45°

Figure 10 presents the variation of the lateral performance of micropiled rafts at different values of vertical loading, where all 36 micropiles were installed inclined with steel casing around steel bar group. It can be seen that increasing vertical loads, causes continuous decrease in the lateral load capacity of micropiled rafts. The maximum lateral load decreases by about 19%, 15%, 22%, and 40% in the case of *F*_*v*_ = 6893 kN compared to the case of pure lateral loading at *θ* = 0°, 15°, 30°, and 45° respectively. When considering the behavior of inclined micropiled rafts under vertical loads in “Pure vertical loads on micropiled raft foundation”, the case of *θ* = 45° seemed to obtain the highest increase rate of vertical displacement with increasing vertical loads. Still here, the case of *θ* = 45° has the highest rate of decrease in the lateral capacity with the increase of vertical loads compared to other values of *θ*.

The lateral capacity reduction with increasing vertical loads may be attributed to the early failure of the micropile-soil interface in the upper soft clay layer under the vertical load action. In this case, the further lateral deformation in the micropile group will not cause increased lateral soil resistance. Hence, this will result in a considerable decrease in the lateral load capacity. This trend is similar to that presented by Rajagopal and Karthigeyan (2008) and Hazzar et al. (2017).

#### Effect of type of micropile reinforcement

**Fig. 11:**
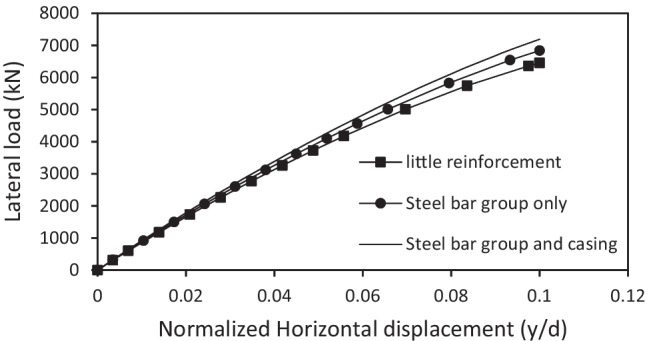
Lateral load displacement response of micropiled raft with reinforcement type of vertical micropiles

**Fig. 12:**
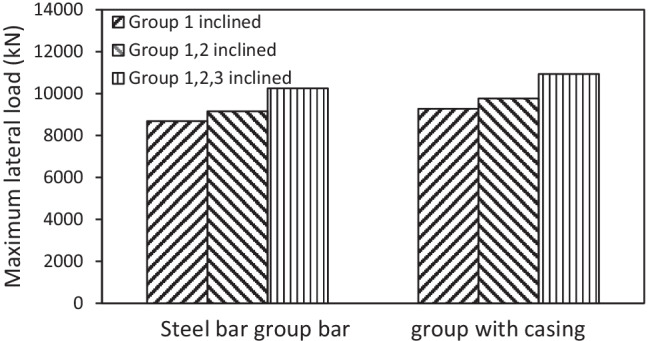
Maximum lateral load taken by micropiled raft with different micropiles reinforcement

The effect of micropile reinforcement type on the lateral response of micropiled raft systems was studied using either steel bar group reinforcement or steel bar group surrounded by a steel casing. This was done in the FEA by changing the micropile modulus of elasticity (see Table 2). Moreover, a single theoretical case was studied with adopting micropile elastic modulus of 30 × 10^6^ kPa to represent micropiles which hardly have any reinforcement. Figure 11 presents the variation in the lateral performance of the micropiled raft with the change in the micropile reinforcement at *F*_*v*_ = 4595 kN where all 36 micropiles were installed vertically. The lateral load capacity increases by only 5% when a casing is placed around the bar group. The lateral response is little dependent on the reinforcement type of the installed micropiles. Figure 12 shows the variation in the maximum lateral load carried by the micropiled raft with the change in the micropile reinforcement at *F*_*v*_ = 4595 kN where all 36 micropiles were installed inclined at *θ* = 30°. It is observed from the figure that the maximum lateral load increased by about 7% in the case of using a permanent casing around the steel bar group compared to the case of steel bar group only. This slight increase can be attributed to increasing the percentage of steel reinforcement by placing an additional steel casing which leads to a higher micropile stiffness. Hence, the lateral load capacity is enhanced.

#### Effect of inclination angle

**Fig. 13:**
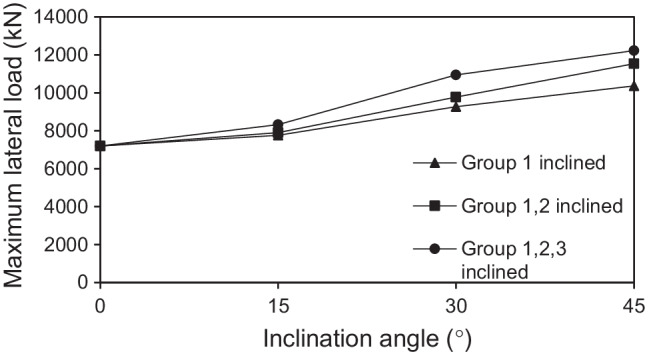
Maximum lateral load taken by micropiled raft with different micropile inclination angles

**Fig. 14:**
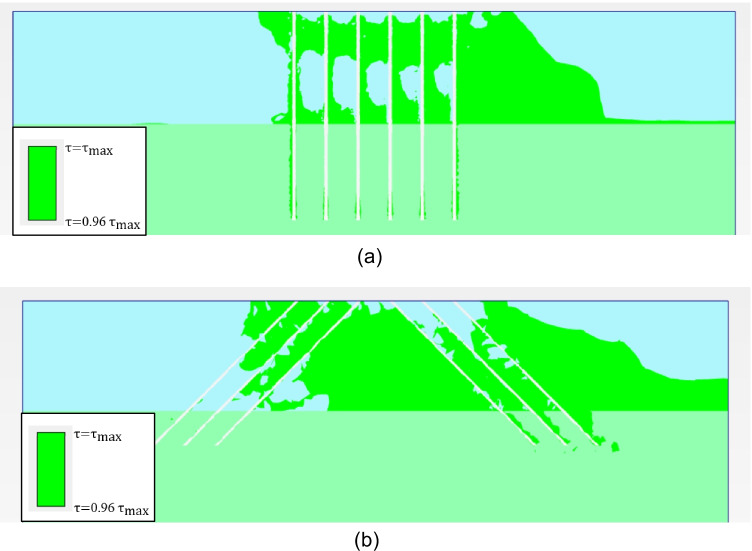
Soil-resisting zones for **a** *θ* = 0^°^ and **b** *θ* = 45^°^

The inclination angle of micropiles, *θ*, represents a significant factor when considering the lateral behavior of micropiled raft. Figure 13 presents the variation of the lateral load capacity of micropiled rafts at different inclination angles in case of using steel case around the steel bar group and *F*_*v*_ = 4595 kN. The figure depicts that the maximum lateral load increases continuously with the increase of *θ*. When all micropiles are inclined, the maximum lateral load increases by about 16%, 52%, and 70% at *θ* = 15°, 30°, and 45°, respectively, compared to the case of *θ* = 0°. The reason for that behavior can be attributed to the increase of the passive resisting zone and skin friction along the micropile surface which both represent the main resisting components for the lateral load–carrying capacity of micropiles (Kyung and Lee 2018). Figure 14 shows the soil-resisting zones along the micropiles of row 3 (see Fig. 7) from the FEA at 0.1 *d* lateral displacement and *F*_*v*_ = 4595 kN. The green color indicates the resisting zones in which the ratio of shear stress to shear strength ranges from 0.96 to unity. A larger green-colored area should mean a larger resisting soil area and longer slip surfaces and, hence, greater lateral load–carrying capacity. It is observed from the figure that most soil-resisting zones were located in the upper soil. Furthermore, the resisting zone for *θ* = 45^°^ in Fig. 14b was greater than that for *θ* = 0^°^ in Fig. 14a by about 40–50%.

#### Effect of number of inclined micropiles

**Fig. 15:**
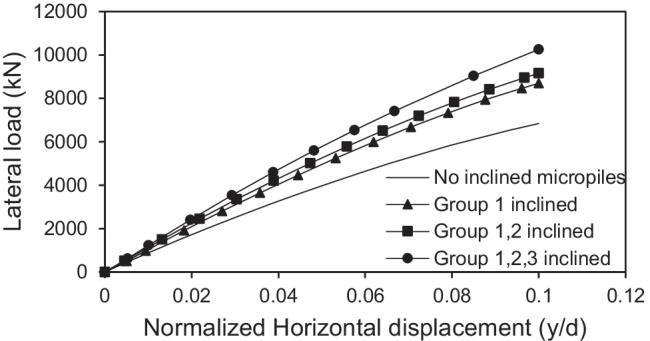
Lateral load displacement response of micropiled raft in case of steel bar group only reinforcement

**Fig. 16:**
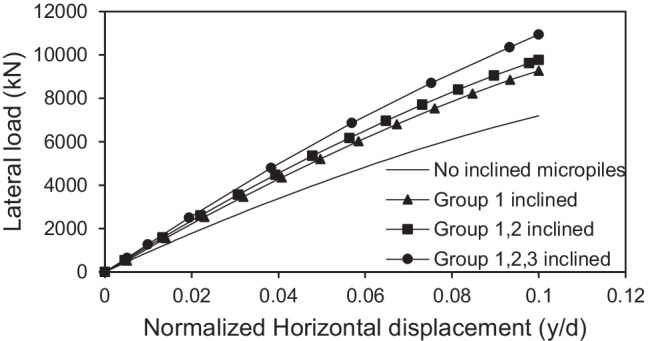
Lateral load displacement response of micropiled raft in case of steel case around the steel bar group reinforcement

The effect of number of inclined micropiles on the lateral response of micropiled raft systems was investigated. Figure 15 presents the variation of the lateral response of micropiled rafts at different numbers of 30° inclined micropiles and *F*_*v*_ = 4595 kN, in which the micropiles were reinforced with steel bar group only. It can be observed that installing more micropiles in an inclined condition enhances the lateral capacity of the micropiled raft. When all the micropiles installed are inclined, the lateral load capacity is 50% higher than the case of no inclined micropiles, 18% higher than the case of installing inclined micropiles of group 1 only, and 12% higher than the case of installing inclined micropiles of groups 1 and 2. A similar trend of variation can be also seen when the micropiles contain steel casing around the steel bar group as shown in Fig. 16.

### Evaluation of the lateral load carried by each micropile in a micropiled raft

When a micropiled group is subjected to combined loads, the micropiles will offer different load capacity depending upon their position and the lateral loading direction. In this study, 36 micropiles were divided in 6 rows with 6 piles in each row. The micropiles on the left-hand side were inclined negatively, whereas the micropiles on the right-hand side were inclined positively (see Fig. 3).

**Fig. 17:**
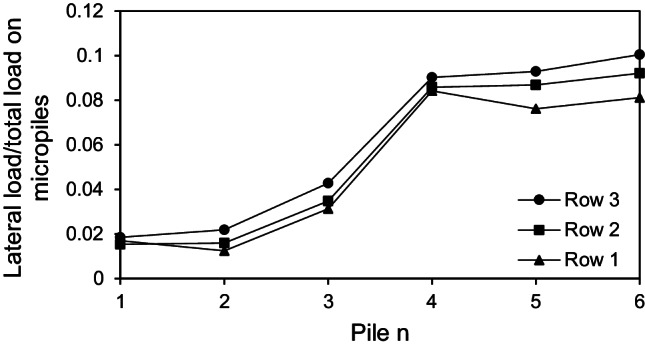
Ratio of lateral load of a micropile to total lateral load carried by micropiles at *θ* = 45°, *F*_*v*_ = 2298 kN

**Fig. 18:**
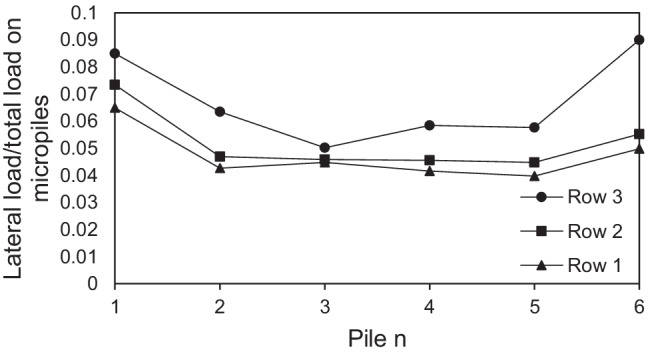
Ratio of lateral load of a micropile to total lateral load carried by micropiles at *θ* = 0°, *F*_*v*_ = 2298 kN

In order to discuss the lateral load distribution among individual micropiles at 0.1 *d* lateral displacement, the ratios of the lateral load of each micropile in the row relative to the total lateral load carried by micropiles are plotted in Fig. 17. The previous analysis was conducted for the case of installing all the micropiles inclined at *θ* = 45°, *F*_*v*_ = 2298 kN and steel casing around the steel bar group. The figure depicted that the lateral loads carried by micropiles subsequently increases from row 1 to row 3, i.e., the row 1 takes the least part of loads while the row 3 carries the largest amount of loads. Furthermore, the positively inclined micropiles (micropiles 4, 5, 6) in the three rows carry much more lateral loads than the negatively inclined micropiles (micropiles 1, 2, 3). The same variation of results has been seen at *θ* = 15°, 30°, and 45°. Comparing all the configurations, it is observed that the positively inclined ones carry almost 79–86% of the total lateral load carried by micropiles, whereas the negatively inclined ones carry 14–21%. This dramatic behavior can be attributed to the micropile inclination direction with respect to the lateral load direction. The micropile position plays an important role as well. It is well known that the leading piles carry more lateral loads than that carried by trailing ones in conventional piled rafts subjected to lateral loads. However, relatively different trend was observed at *θ* = 0° as shown in Fig. 18. The load distribution among the vertical micropiles tends to be more uniform compared to the inclined cases and the most trailing micropile seems to carry the highest lateral load in row 2 and row 1. This different behavior can be attributed to the absence of micropile inclination and the presence of vertical loading which may help redistribute the lateral loads among the micropiles compared to the case of pure lateral loading. These findings are similar to that presented by Hazzar et al. (2017).

**Fig. 19:**
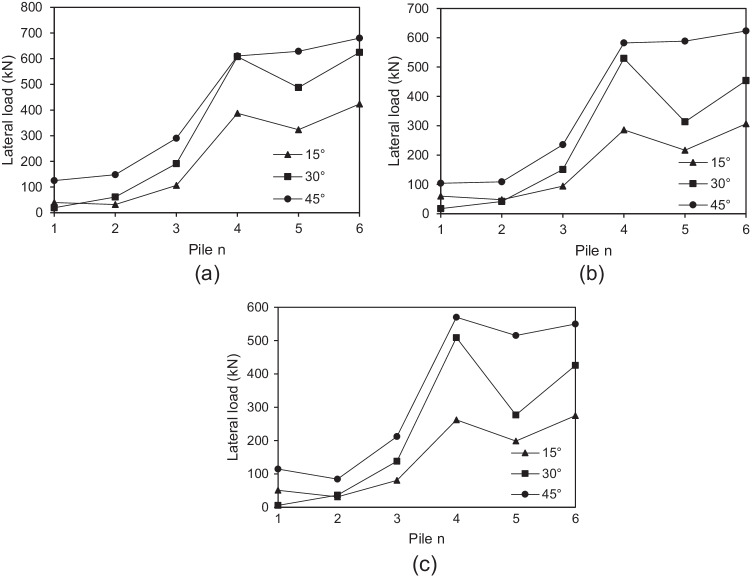
Lateral load value carried by each individual micropile at various *θ* values, *F*_*v*_ = 2298 kN (**a**) row 3 (b) row 2 (**c**) row 1

In order to discuss the effect of micropile inclination angle on the load distribution among individual micropiles at 0.1 *d* lateral displacement, the absolute values of lateral loads taken by the inclined micropiles are plotted in Fig. 19 at different values of *θ*, *F*_*v*_ = 2298 kN and steel casing around the steel bar group. It can be observed that the lateral load carried by each individual micropile increases with the increase of *θ*, which of course helps to improve the lateral resistance of micropiled rafts when *θ* is increased as discussed in “Effect of inclination angle.”

### Evaluation of load sharing ratio between micropiles and raft

Applied loads are shared by both the raft and micropiles connected to the raft. A part of the load is carried by the raft and rest of the load is carried by the micropiles. Installing micropiles with inclined condition would lead to a further complicated sharing behavior. According to the current design practice of conventional pile foundations, piles are designed assuming that they take completely the total value of both applied vertical loads and horizontal loads leading to a high number of piles and a very safe foundation system but very expensive. Therefore, there is a need to assess this complex load sharing nature by evaluating the load sharing ratio between the micropiles and the raft leading to more economical foundation system design.

The lateral load sharing ratio (α_h_) can be defined as the proportion of the lateral load taken by the micropiles to the total lateral load taken by the micropiled raft while the vertical load sharing ratio (α_v_) can be defined as the proportion of the vertical load taken by the micropiles to the total vertical load taken by the micropiled raft.

**Fig. 20:**
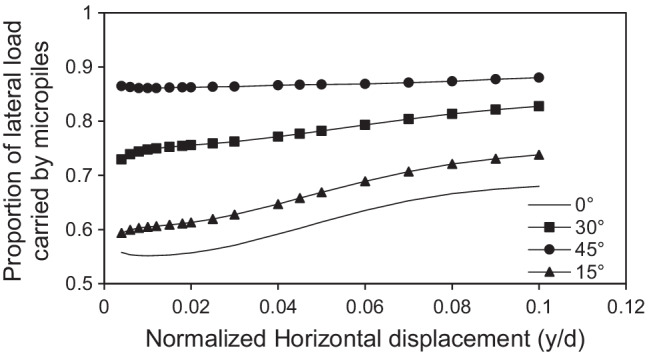
Load sharing ratio of micropiled rafts with lateral displacement at various values of *θ*

Figure 20 shows the proportion of the lateral load carried by the inclined micropiles with normalized horizontal displacement (y/d) at different values of *θ*, *F*_*v*_ = 2298 kN and steel casing around the steel bar group. It can be seen that there is a nonlinear relationship between *α*_*h*_ and y/d. The value of *α*_*h*_ tends to be displacement-dependent and slightly increase with the increase of lateral displacement, as the raft transfers the load to the micropiles. The case of *θ* = 15° shows the highest rate of load increase, where *α*_*h*_ increases from 59 to 74% when y/d increases from 0.004 to 0.1. On the other hand, the case of *θ* = 45° shows the lowest rate of load increase, where *α*_*h*_ increases by only 2% when y/d increases from 0.004 to 0.1.

**Fig. 21:**
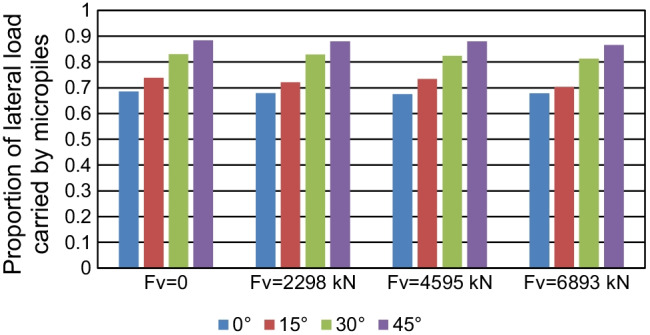
Lateral load proportion carried by micropiles at 0.1 *d* lateral displacement at different *θ* values

**Fig. 22:**
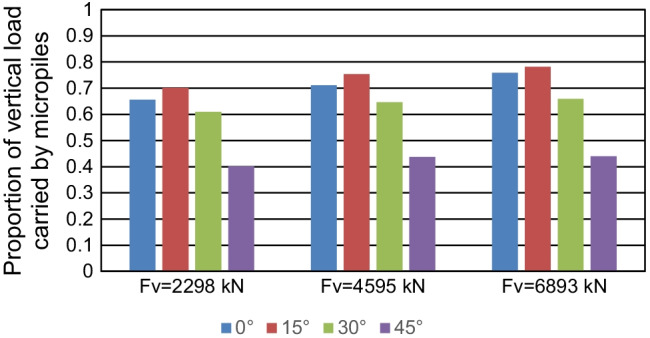
Vertical load proportion carried by micropiles at 0.1 *d* lateral displacement at different *θ* values

The impact of both micropile inclination angle and magnitude of vertical loading in controlling *α*_*h*_ and *α*_*v*_ ratios was checked. Figure 21 presents the values of *α*_*h*_ with different values of micropile inclination angle, whereas Fig. 22 presents the values of *α*_*v*_ with different values of micropile inclination angle, where all the micropiles were installed inclined with a steel case around the steel bar group. The figure depicts that *α*_*h*_ and *α*_*v*_ are dependent on the micropile inclination angle to a great extent. Inclined micropiles offer greater *α*_*h*_ than that of vertical ones, largest at *θ* = 45°. For example, at *F*_*v*_ = 2298 kN, *α*_*h*_ increases from 68 to 88% when *θ* is changed from 0 to 45°. However, an opposite trend takes place when considering *α*_*v*_ which seems to increase initially when *θ* increases from 0 to 15°, then it decreases continuously with increasing *θ*. This variation trend of *α*_*v*_ with *θ* is consistent with the results by Kim et al. (2018) who confirmed that the maximum *α*_*v*_ value of inclined micropiled rafts is obtained at *θ* = 15°.

The magnitude of vertical loading has a little effect on *α*_*h*_. The highest variation in *α*_*h*_ is at *θ* = 15°, where it decreases by only 3.6% when *F*_*v*_ increases from 0 to 6893 kN. However, *F*_*v*_ has a relatively higher impact on the values of *α*_*v*_. For vertical micropiles, *α*_*v*_ increases from 66% to 76% when *F*_*v*_ increases from 2298 kN to 6893 kN. For *θ* = 15°, *α*_*v*_ increases from 70 to 78% when *F*_*v*_ increases from 2298 kN to 6893 kN. A less increase in *α*_*v*_ is observed with increasing *F*_*v*_ at *θ* = 30°, 45°. Comparing all the configurations, it is observed that the raft carries 24–60% of vertical load, whereas the micropiles carry 40–76%. Regarding the applied lateral load, the load carried by the raft varies between 12 and 32% of the total lateral load, while the micropiles carry 68–88%.

### Percentage of increase in lateral load capacity

In order to check the influence of installing inclined micropiles against using additional steel casing on the lateral response of micropiled rafts, the percentage of increase in the lateral load capacity was evaluated. The percentage of increase can be defined as

3 $$\mathrm{IL}=\frac{{\mathrm{L}}_{\mathrm{wi}}-{\mathrm{L}}_{\mathrm{ni}}}{{\mathrm{L}}_{\mathrm{ni}}}\ast 100$$

where IL, percentage of increase in lateral load capacity with inclined micropiles; L_wi_, lateral load capacity with inclined micropiles (kPa); L_ni_, lateral load capacity with vertical micropiles (kPa).

**Fig. 23:**
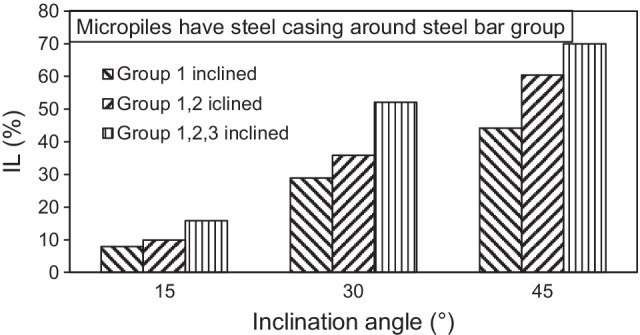
Percent increase in the lateral load capacity for different inclination angles of micropiles at various number of inclined micropiles and *F*_*v*_ = 4595 kN

**Fig. 24:**
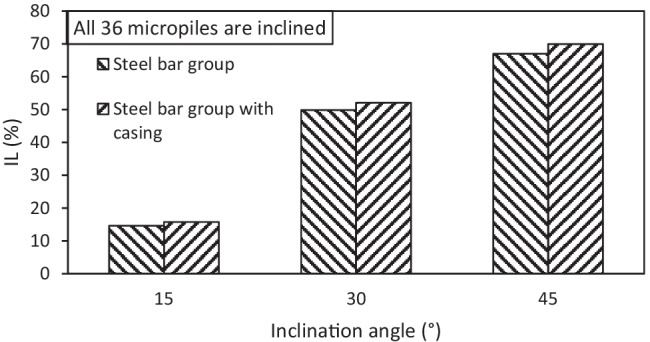
Percent increase in the lateral load capacity for different inclination angles of micropiles at various reinforcement types and *F*_*v*_ = 4595 kN

Figure 23 presents the percent increase in the lateral load capacity for different inclination angles of micropiles at various number of inclined micropiles and *F*_*v*_ = 4595 kN, whereas Fig. 24 presents the percent increase in the lateral load capacity for different inclination angles of micropiles at various reinforcement types and *F*_*v*_ = 4595 kN. The figures depict that values of IL increase with the increase of inclination angle of installed micropiles, i.e., the inclination angle has a positive impact on IL. Furthermore, IL also increases with increasing the number of inclined micropiles. However, it is observed that the effect of micropiles reinforcement on IL is low compared to that of micropile inclination angle. This finding is similar to that presented by El Kamash and Han (2017) who stated that the performance of floating micropiles under vertical loads seemed to be little affected by the micropile elastic modulus.

## Conclusions

The performance of micropiled rafts installed with inclined condition under combined loading has been studied using a series of 3D finite element numerical analyses. The main conclusions drawn from the numerical results are listed below:

1. Inclined micropiled rafts offer the highest resistance to vertical loads at *θ* = 15° followed by a gradual decrease in the resistance with further increase of *θ*.
2. Increasing vertical loads causes continuous decrease in the lateral load capacity of micropiled rafts, with the highest rate of decrease at the case of *θ* = 45° compared to other values of *θ*.
3. The reinforcement of the micropiles supporting the micropiled raft has a relatively little effect on its lateral response. The lateral load capacity increases slightly with placing a permanent steel casing around the steel bar(s) reinforcement.
4. The lateral load capacity is much dependent on the micropile inclination angle and increases continuously with the increase of *θ* up to *θ* = 45^°^. This can be attributed to the increase of the passive resisting zone and skin friction along the micropile surface.
5. Increasing the number of inclined micropiles enhances the lateral performance.
6. For the studied micropiled raft, when all micropiles installed are inclined, the positively inclined micropiles carry 79–86% of the total lateral load carried by micropiles, whereas the negatively inclined ones carry 14–21%. This can be attributed to the micropile inclination direction with respect to the lateral load direction. The micropile position plays an important role as well. However, relatively different trend was observed at *θ* = 0°. The lateral load distribution among the vertical micropiles tends to be more uniform compared to the inclined cases.
7. There is a nonlinear relationship between lateral load sharing ratio and lateral displacement. The value of *α*_*h*_ is displacement-dependent and slightly increases with the increase of lateral displacement, as the raft transfers the load to the micropiles. The case of *θ* = 15° shows the highest rate of micropile load increase with lateral displacement.
8. The lateral and vertical load sharing ratios at 0.1 *d* lateral displacement greatly depend on the inclination angle of micropiles. Inclined micropiles offer greater *α*_*h*_ than that of vertical ones, largest at *θ* = 45°. However, an opposite trend takes place when considering *α*_*v*_ which seems to increase initially when *θ* increases from 0 to 15°, then it decreases continuously with increasing *θ*.
9. The percentage of increase in the lateral load capacity is evaluated. The inclination angle has a positive impact on IL. Furthermore, IL also increases with increasing the number of inclined micropiles. However, it has been observed that the effect of micropile reinforcement on IL is low compared to the effect of micropile inclination angle.
10. Considering the findings of this paper, design engineers are encouraged to install combined inclined and vertical micropiles under the same raft, which could help optimize the design of micropiled rafts in terms of both vertical and lateral performance.

## Data Availability

All data, models, and code generated or used during the study appear in the submitted article.
